# Risk Factors and Outcome for Massive Intra-Abdominal Bleeding Among Patients With Infected Necrotizing Pancreatitis

**DOI:** 10.1097/MD.0000000000001172

**Published:** 2015-07-17

**Authors:** Xiao Shen, Jing Sun, Jingzhu Zhang, Lu Ke, Zhihui Tong, Gang Li, Wei Jiang, Weiqin Li, Jieshou Li

**Affiliations:** From the Department of General Surgery, Surgical Intensive Care Unit (SICU) (XS, JZ, LK, ZT, GL, WJ, WL); and Department of General Surgery, Jinling Hospital, Medical School of Nanjing University, No. 305 Zhongshan East Road, Nanjing, Jiangsu Province, China (JS, JL).

## Abstract

The incidence of acute bleeding is reported to be 13.5% in patients with acute necrotizing pancreatitis. However, of all the bleeding events, intra-abdominal bleeding was less studied in the literature and its risk factors have not been well defined yet. The purpose of the present study was to investigate the risk factors for massive intra-abdominal bleeding among the patients with infected necrotizing pancreatitis and assessed the outcome of these patients.

Both univariate and multivariate logistic regression models were applied for evaluating risk factors for intra-abdominal bleeding using 33 indices, including age, sex, etiology of acute pancreatitis (AP), APACHE II score, etc. Outcome assessments such as mortality, hospital and intensive care unit (ICU) durations, and cost were also compared between patients with or without intra-abdominal bleeding.

Acute kidney injury (AKI) (odds ratio [OR]: 7.54, 95% confidence interval [CI]: 2.53–22.52, *P* < 0.001) and number of operation (OR: 8.84, 95% CI: 2.01–38.86, *P* = 0.004) were 2 predictors for massive intra-abdominal bleeding in the patients with infected necrotizing pancreatitis. In addition, AP patients with intra-abdominal bleeding also showed significantly higher mortality rate, prolonged hospital and ICU durations, more complications and invasive treatments, as well as increased cost.

Our study revealed that AKI and multiple operations were 2 critical factors increasing the risk of intra-abdominal bleeding among patients with infected necrotizing pancreatitis. Additionally, massive intra-abdominal bleeding was also associated with poor prognosis.

## INTRODUCTION

Acute pancreatitis (AP) is an inflammatory disorder of the pancreas, characterized by edema, autodigestion, fat necrosis, and hemorrhage of pancreatic tissue. Around 5% to 10% of the AP patients would develop necrosis, either sterile or infected, of the pancreatic and/or peripancreatic tissue.^1^ Infected pancreatic necrosis (IPN) has been proved to be a definite determinant of mortality in AP patients.^2^

Acute bleeding is a relatively rare but severe complication of AP.^3,4^ The incidence of acute bleeding is reported to be 13.5% in patients with necrotizing pancreatitis, much higher than 1.5% in those with interstitial pancreatitis.^5^ The major causes for the bleeding include peptic ulcers, gastric, duodenal or colonic lesions, regional portal hypertension-induced gastric varices, etc.^5^ Of all the bleeding events, gastrointestinal (GI) bleeding, once regarded as a type of organ failure, was the mostly concerned one.^6^ Intra-abdominal, also called intra-abdominal hemorrhage, is another common complication of AP and other abdominal disease.^7–9^ It may cause a series of complications such as hemorrhagic shock, cerebral infarction, and abortion and lead to a poor prognosis.^8,10^ However, intra-abdominal bleeding in the case of AP was less studied in the literature. A study conducted by Flati et al^11^ reported that massive intra-abdominal bleeding associated with major vessel injury was considered as the primary cause for >50% of the death cases of the AP patients. Unfortunately, they did not make any further studies after that and the risk factors for intra-abdominal bleeding among AP patients have not been well defined yet, making early preventive interventions unlikely.

The purpose of the present study was to investigate multiple potential risk factors for massive intra-abdominal bleeding in the patients with infected necrotizing pancreatitis as well as to assess the prognosis of the AP patients with intra-abdominal bleeding.

## MATERIALS AND METHODS

### Subjects

This is an observational study performed at Jinling Hospital, Medical School of Nanjing University. Patients with a primary diagnosis with AP admitted to the Surgical Intensive Care Unit (SICU), Department of General Surgery during the study period between January 2010 and December 2013 were screened for potential recruitment. Inclusion criteria were AP patients aged 18 to 75 years, with a confirmed diagnosis of IPN. Patients were excluded if they were in pregnancy, have received operative surgery before admission for necrosectomy, abdominal compartment syndrome (ACS), etc. or developed intra-abdominal bleeding before admission during the current episode of AP. Patients were divided into 2 groups according to the presence of massive intra-abdominal bleeding: bleeding group, in which patients suffered massive intra-abdominal bleeding during the course of AP; nonbleeding group, in which patients did not suffer massive intra-abdominal bleeding during the course of AP. The study was approved by the institutional review board of Jinling Hospital.

### Definition

The definition of AP was according to the Atlanta criteria.^1,12^ IPN was diagnosed when ≥1 of the following criteria was met: the presence of extraluminal gas in the pancreatic and/or peripancreatic tissues on contrast-enhanced computed tomography (CECT); a positive bacterial culture of the necrotic tissue from the fine-needle aspiration or drainage.^1^ Massive intra-abdominal bleeding was defined as significant hemodynamic deterioration and/or a sharp decrease in hemoglobin concentration of >2 g/dL caused by the bleeding event in the abdomen during the course of or following AP.^11^ Organ failure was assessed and defined on 4 organ systems: respiratory, renal, cardiovascular, and hepatic. Respiratory failure was defined as PaO_2_/FiO_2_ ≤ 300 mmHg (40 kPa), renal failure was defined as serum creatinine ≥171 μmol/L (2.0 mg/dL) and cardiovascular was defined as systolic blood pressure <90 mmHg or need for inotropic agent according to a recently published international consensus.^13^ The criteria for hepatic failure was defined as a score of ≥2 using the Marshall scoring system.^14^ The definition of sepsis was according to the presence of infection as well as the systemic manifestations on the basis of the surviving sepsis campaign (SSC) 2012.^15^ According to the recently updated consensus definition of the World Society of Abdominal Compartment Syndrome (WSACS), ACS was defined as a sustained intra-abdominal pressure (IAP) >20 mmHg (with/without an abdominal perfusion pressure [APP] <60 mmHg) associated with new-onset organ dysfunction/failure.^16^ Multiple operations were defined as ≥2 operations on the same patient during the current episode of AP for necrosectomy, ongoing ACS, or GI fistula, etc. Daily fluid balance was measured as the difference between total fluid input and total fluid output in the same 24-hour period. Insensible fluid losses were calculated according to Dubois equation^17^:

Body surface area = 71.84 × (body weight in kilogram)^0.425^ × (height in centimeter)^0.725^ (1)

Insensible fluid loss = 550 ml / body surface area (2)

### Data Collection

After admission, the demographic characteristics (including age, gender, height, weight, etc.) of each patient were collected and recorded. Body mass index (BMI) was calculated as the weight (in kilograms) divided by the square of the height (in meters). Patients were assessed with blood routine and biochemical tests and evaluated with CECT during the first 24 hours after admission. Initial vital signs such as body temperature and blood pressure were monitored and recorded every 4 hours in the intensive care unit (ICU). IAP was measured according to the standard procedure of WSACS, using a catheter inserted into the bladder every 6 hours for the first 24 hours. Based on the demographic characteristics, the laboratory results and the organ functions, the Acute Physiology and Chronic Health Evaluation (APACHE) || score and Sequential Organ Failure Assessment (SOFA) score were assessed and recorded. Computed tomography (CT) severity index was evaluated according to the image findings based on the Balthazar's CT score. Daily fluid balance was recorded in detail. Outcome assessments including mortality, hospital and ICU durations, incidence of multiple organ failure (MOF) and positive blood culture, requirement for invasive treatments, as well as cost were collected for prognostic analysis.

### Statistical Analysis

SPSS 22.0 statistical software package (IBM Analytics, Armonk, NY) was applied for statistical analyses. Data are presented as median plus interquartile range (IQR) for continuous variables and absolute numbers and percentages for categorical variables. Student *t* test or Mann–Whitney test was used for analyzing continuous variables and the *χ*^2^ test was used for analyzing categorical variables. To identify the potential risk factors for massive intra-abdominal bleeding, univariate logistic regression analysis for aforementioned 33 indices was performed. Variables presenting statistical significance in univariate logistic analyses were included for further multiple logistic regression analysis. Odds ratios (ORs) are expressed with 95% confidence intervals (CIs). Statistical significance was considered as a *P* value of <0.05 (2-tailed).

## RESULTS

### Baseline Characteristics and Clinical Features

After screening, a total of 288 patients with infected necrotizing pancreatitis were included for analysis in this study. Of which, 84 patients (29.2%) suffered massive intra-abdominal bleeding during hospitalization. The baseline characteristics of the AP patients with/without massive intra-abdominal bleeding were displayed in Table 1. Two hundred and seven (207/288, 71.9%) patients were transferred from other hospital and the median time interval from AP onset to admission was 28 (IQR: 11–53) days for all the patients. The median age of the study patients was 46 (IQR: 37–75) years, predominantly male patients. Patients in the bleeding group had significantly higher APACHE II and SOFA scores. Around three-fourths of the bleeders and half of the non-bleeders had an extent of pancreatic necrosis >50% (61/84 [72.6%] vs 110/204 [53.9%], *P* = 0.003).

**TABLE 1:**
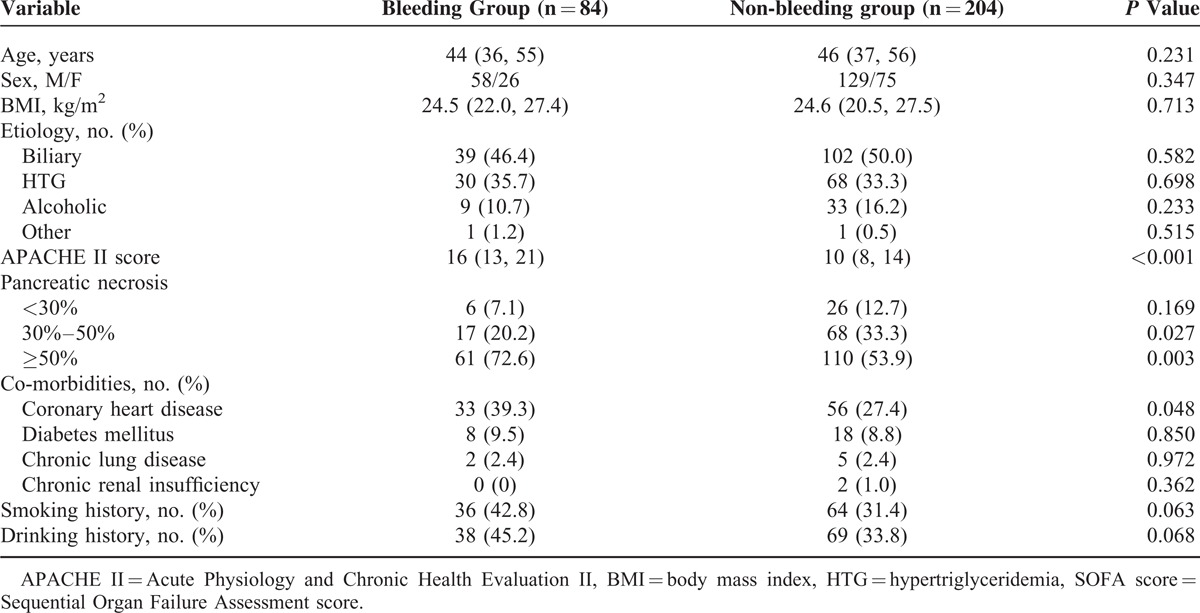
Baseline Characteristics of the Patients With/Without Massive Intra-abdominal Bleeding

Table 2 showed the bleeding sources of the bleeders as well as the management they received for treatment. Concerning the bleeding sites, the highest mortality was observed when the bleeding event occurred via external drainage catheters after necrosectomy (2/2, 100%) or after retroperitoneal bleeding (26/40, 65.0%) or after splenic ruptures (3/6, 50.0%), which was consistent with previous study.^11^ In addition, gastric artery was one of the mostly involved vessels in the intraperitoneal bleeding. Soon after bleeding, resuscitation was allowed in every patient with massive intra-abdominal bleeding. Preoperative angiographic embolization and/or packing was attempted at the same time, and in 33 (33/84, 39.3%) cases that needed further operation, the angiographic and/or packing control was only temporary.

**TABLE 2:**
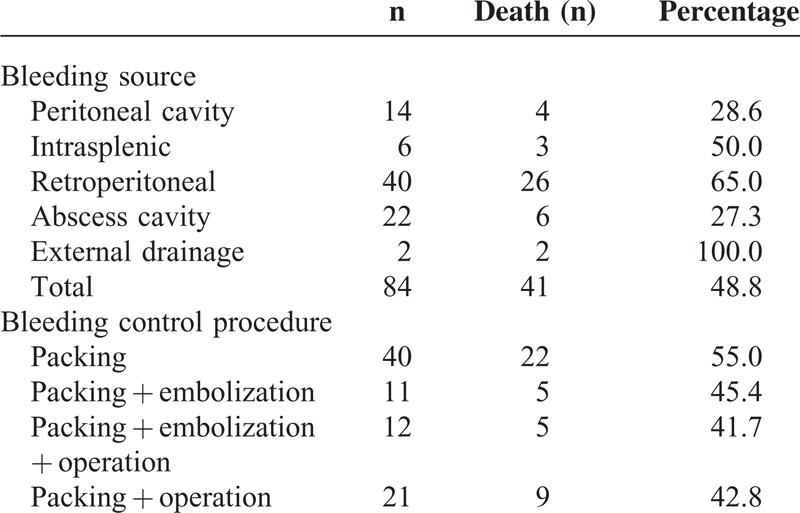
Bleeding Sites and Management for the Patients With Massive Intra-abdominal Bleeding

### Risk Factors Predicting Massive Intra-Abdominal Bleeding

**FIGURE 1:**
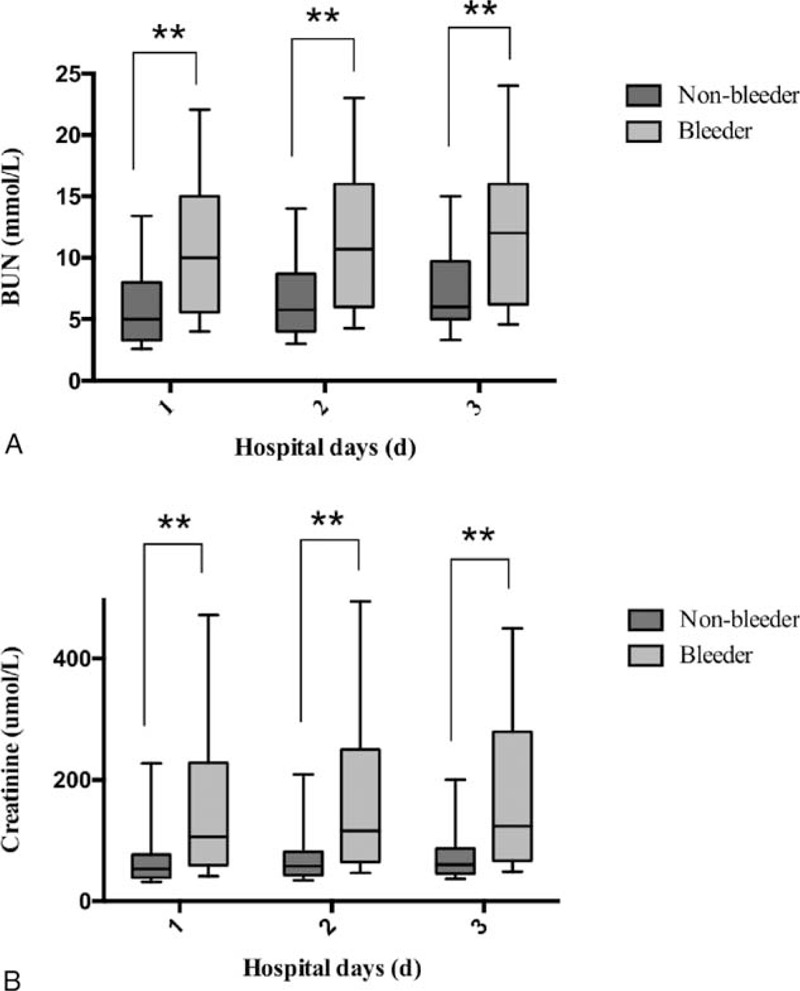
Serum levels of blood urea nitrogen (BUN) and serum in the pancreatitis patients with or without massive intra-abdominal bleeding. (A) Serum levels of BUN in the pancreatitis patients within the first 3 days after admission. (B) Serum levels of creatinine in the pancreatitis patients within the first 3 days after admission.

Comparison between AP patients with/without massive intra-abdominal bleeding indicated that there were significant differences between the 2 groups in the following aspects (Table 3): time from AP onset to admission, temperature, IAP, total and direct bilirubin, blood urea nitrogen (BUN), creatinine, platelet count, pathogen for IPN, organ failure, sepsis and ACS on admission, number of operation as well as time interval from AP onset to operation. The median BUN of the patients with massive intra-abdominal bleeding on admission was significantly higher than those without (10.2 [IQR: 5.9–16.0] mmol/L vs. 5.0 [IQR: 3.3–8.4] mmol/L, *P* < 0.001) and this tendency also sustained for the following two days after admission (Figure 1A). Similar trends were also seen in serum creatinine (Figure 1b).

**TABLE 3:**
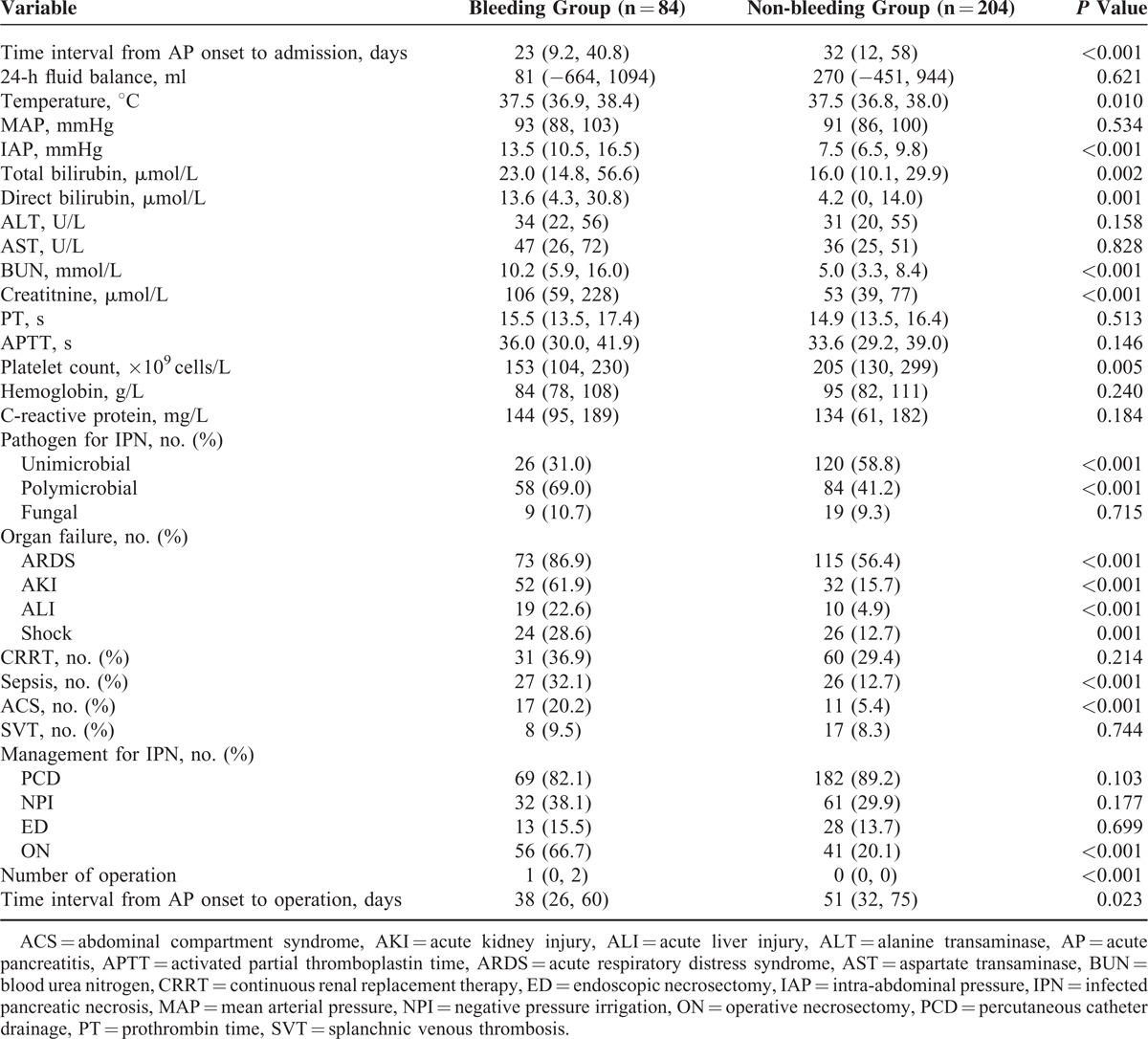
Comparison of the Patients With/Without Massive Intra-abdominal Bleeding

Univariate logistic regression analysis including 33 indices (Table 4) revealed significant correlations between massive intra-abdominal bleeding and time interval from AP onset to admission, APACHE II score, SOFA score, CT severity index, IAP, total and direct bilirubin, BUN, creatinine level and platelet count on admission, pathogen for IPN, organ failure, shock, sepsis and ACS on arrival, performance and number of operation as well as time interval from AP onset to operation. Further multivariate regression analysis taking all the significant variables in univariate regression model indicated that AKI (OR: 7.45, 95% CI: 2.53–22.52, *P* < 0.001) and number of operation (OR: 8.84, 95% CI: 2.01–38.86, *P* = 0.004) were 2 independent risk factors for massive intra-abdominal bleeding.

**TABLE 4:**
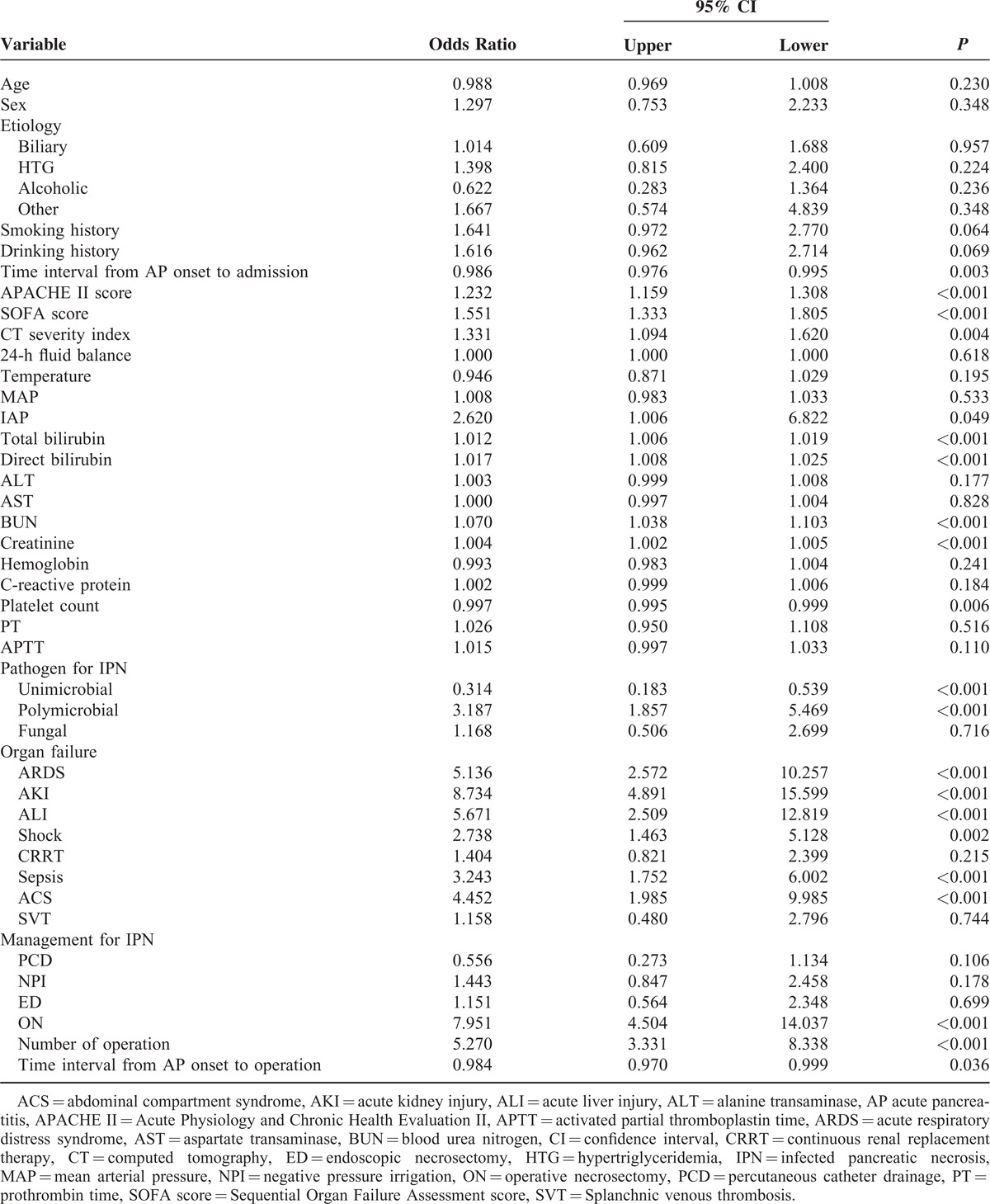
Univariate Logistic Regression Analysis for Massive Intra-abdominal Bleeding

### Prognosis and Outcome

Table 5 presented with a variety of clinical variables regarding the outcome of the patients with/without massive intra-abdominal bleeding. Forty-one (48.8%) of the 84 patients with massive intra-abdominal bleeding died during hospitalization. Septic shock and consequent MOF were the major death cause for most of non-survivors. Also, it was notable that recurrent bleeding-related MOF accounted for >20% of the death cases. In the non-bleeding group, the mortality rate was markedly lower, only 17.2% (41/84 [48.8%] vs 35/204 [17.2%], *P* < 0.001). Of which, septic shock and consequent MOF were the death cause for almost all the non-survivors. The incidence of organ failure in infected necrotizing pancreatitis patients was quite high during the clinical course: respiratory failure in 196 patients (68.0%), cardiovascular failure in 95 patients (33.0%), renal failure in 101 patients (35.1%), and hepatic failure in 45 patients (15.6%). Furthermore, the prolonged ICU and hospital durations as well as the increased cost also indicated worse outcomes of the patients with intra-abdominal bleeding when compared with those without. Fifty-six (66.7%) of the 84 bleeding patients and 41 (20.1%) of the 204 non-bleeding patients received operative surgery. Confirmed IPN or GI fistula was the indication for the primary surgical intervention for all the surgical patients in the non-bleeding group and 36 surgical patients in the bleeding group (77/97, 79.4%). The remaining 20 patients required surgical intervention for ongoing ACS or bleeding control.

**TABLE 5:**
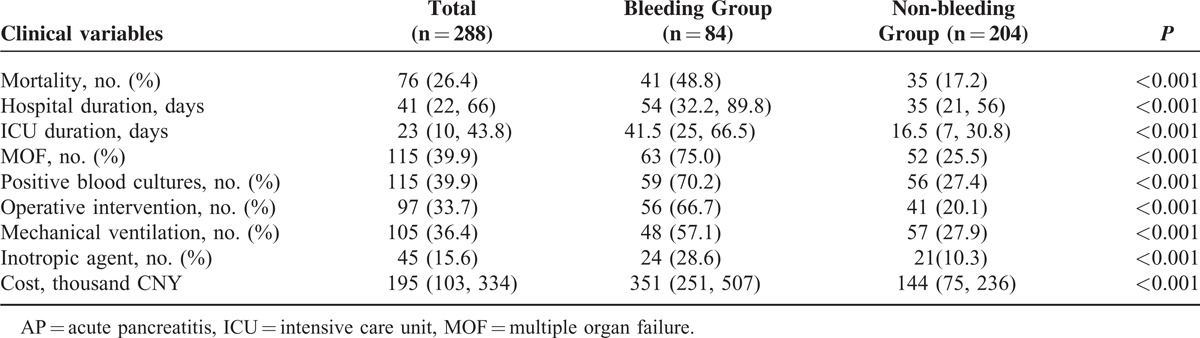
Clinical Course and Outcome of AP Patients With or Without Massive Intra-abdominal Bleeding

## DISCUSSION

Acute bleeding, a lethal complication of AP, is associated with a high mortality rate up to 52.4% and contributes to 12.5% of the fatal outcomes in AP patients in the literature.^4,18,19^ It was reported that GI bleeding was more common than intra-abdominal bleeding, contributing to 69% and 31% of the bleeding events in pancreatic diseases, respectively.^20^ Whereas, according to our experience, the incidence of massive intra-abdominal bleeding in the patients with severe acute pancreatitis (SAP) was around 30%, much higher than GI bleeding (1%). Moreover, different from other situations, intra-abdominal bleeding in AP patients could deteriorate pre-existing organ dysfunction and coagulation disorder and increase the risk of death. In our study, the mortality rate of those patients with massive intra-abdominal bleeding was up to 50%.^11^ Therefore, it is of vital importance to identify the population at high bleeding risk and make early preventive interventions. In the present study, we first evaluated the risk factors for massive intra-abdominal bleeding among the patients with infected necrotizing pancreatitis and demonstrated that AKI and number of operations were 2 independent prognostic factors. Additionally, our data also indicated that AP patients with massive intra-abdominal bleeding were always associated with higher mortality rate, longer hospital duration, as well as more complications.

Timing of surgery, surgical technique, and management strategies including number of operations were already regarded as critical factors that affect the bleeding event in the clinical course of AP since decades ago.^11,21^ Unfortunately, none of the studies has ever systemically investigated the independent risk factors for intra-abdominal bleeding in AP population. So far, our study was the first study that screened the risk factors for massive intra-abdominal bleeding in AP patients and confirmed that number of operation was truly an independent risk factor for massive intra-abdominal bleeding. IPN was also regarded as an important factor affecting the bleeding event in the pancreatitis patients. Whereas, owing to the fact that the subjects were patients with infected necrotizing pancreatitis in the present study, this factor was beyond our consideration.

Clinical bleeding is a major manifestation of renal failure in the pre-dialysis era.^22^ However, the exact mechanism of bleeding in uremic condition was not clear yet.^23^ Multifactorial defect of the interaction between vessel walls and circulating cells seems to be one of the most popular theories.^24–26^ In accordance with previous studies, our study also suggested that AKI was an independent risk factor for massive intra-abdominal bleeding in IPN patients. Although hemodialysis could partially improve the renal function as well as reduce the bleeding risk, the anticoagulant agents used during hemodialysis could further increase the risk of bleeding.^27^ In this way, the presence of AKI would inevitably contribute to the bleeding event. At the same time, it was also notable that BUN and serum creatinine, the indicators for renal function, were not proved to be effective risk factors for intra-abdominal bleeding. This might attribute to the influence of renal replacement therapy on the 2 parameters.

More recently, Zhan et al^28^ investigated the risk factors for upper GI bleeding (UGIB) in SAP patients and suggested that APACHE II score, CT severity index, and PaO2 were independent risk factors for UGIB. However, they had not brought AKI into their analysis. Worse still, they even left the presence of splanchnic venous thrombosis, one of the most important causes for regional portal hypertension and esophagogastric varices in SAP courses, out of their analysis, which might bring in substantial uncertainty to the results.^29,30^

There are also some limitations in our study though. First, due to the retrospective nature of this study, there exists some selection bias for the study. Second, as our center is a tertiary referral one, a good part of patients who developed intra-abdominal bleeding before admission were excluded. Lastly, the number of the bleeders was relatively small and this might affect the precision of our analysis. However, we thought it would still leave AKI and number of operations as valuable tools for predicting intra-abdominal bleeding.

## CONCLUSION

In the present study, we investigated the potential risk factors for massive intra-abdominal bleeding in the patients with infected necrotizing pancreatitis and revealed that AKI and multiple operations were 2 factors increasing the risk of intra-abdominal bleeding. In addition, pancreatitis patients with massive intra-abdominal bleeding were always associated with poor prognosis.
